# Daily Variation in Nursing Home Staffing and Its Association With Quality Measures

**DOI:** 10.1001/jamanetworkopen.2022.2051

**Published:** 2022-03-14

**Authors:** Dana B. Mukamel, Debra Saliba, Heather Ladd, R. Tamara Konetzka

**Affiliations:** 1 Department of Medicine, Division of General Internal Medicine, University of California, Irvine; 2 UCLA Borun Center at David Geffen School of Medicine, Los Angeles, California; 3 Veterans Administration GRECC, Los Angeles, California; 4 RAND Health, Santa Monica, California; 5 Department of Public Health Sciences, The University of Chicago, Chicago, Illinois

## Abstract

**Importance:**

Average staffing measures are a focus of nursing homes’ quality assessments and reporting. They may, however, mask daily variation in staffing, additional information that could be important for understanding nursing home quality and relative ranking.

**Objective:**

To examine daily variation in staffing, its association with quality, and whether daily variation provides information regarding quality ranking of nursing homes over and above the information provided by average staffing levels.

**Design, Setting, and Participants:**

This quality improvement study included registered nurses (RNs) and certified nurse aide (CNAs) at 13 339 certified nursing homes throughout the United States during 2017 to 2018. Retrospective analyses of the Payroll-Based Journal, Medicare Cost Reports, and Nursing Home Care Compare were conducted. Data were analyzed from January 2017 to December 2018.

**Main Outcomes and Measures:**

Three measures of daily variation, ie, coefficient of variation (COV), total outlier days (TOD), and low outlier days (LOD), were calculated for RNs and CNAs. The association between these measures and quality rankings and other facility characteristics were evaluated.

**Results:**

A total of 13 339 nursing homes were included in this study, with 9476 (71%) for-profit facilities. The mean (SD) hours-per–resident-day were 0.41 (0.29) for RNs and 2.16 (0.49) for CNAs, and a mean (SD) 55% (26%) of residents were Medicaid beneficiaries. Outcome measures were as follows: mean (SD) COV, 0.5 (0.6) for RNs and 0.1 (0.1) for CNAs; mean (SD) TOD, 220 (69) for RNs and 44 (45) for CNAs; and mean (SD) LOD, 116 (45) for RNs and 22 (24) for CNAs. All 3 variation measures, for both RNs and CNAs, were significantly associated with both the 5-Star Quality Measures (COV among RNs, −0.014 [95% CI, −0.021 to −0.007]; *P* < .001; COV among CNAs: −0.004 [95% CI, −0.006 to −0.003]; *P* < .001; TOD among RNs, −3.79 [95% CI, −4.59 to −2.99]; *P* < .001; TOD among CNAs, −2.52 [95% CI, −3.08 to −1.96]; *P* < .001; LOD among RNs, −2.46 [95% CI, −3.03 to −1.88]; *P* < .001; LOD among CNAs, −1.29 [95% CI, −1.58 to −0.99]; *P* < .001) and the 5-Star Survey rankings (COV among RNs,−0.026 [95% CI, −0.033 to −0.019]; *P* < .001; COV among CNAs: −0.006 [95% CI, −0.007 to −0.004]; *P* < .001; TOD among RNs, −5.10 [95% CI, −5.97 to −4.23]; *P* < .001; TOD among CNAs, −4.16 [95% CI, −4.77 to −3.55]; *P* < .001; LOD among RNs, −3.04 [95% CI, −3.65 to −2.44]; *P* < .001; LOD among CNAs, −1.97 [95% CI, −2.29 to −1.65]; *P* < .001) published in Nursing Home Care Compare. Low κ values, ranging from 0.23 to 0.63, indicated that the variation measures add information about ranking to the information provided by average staffing measure.

**Conclusions and Relevance:**

These findings highlight the importance of reporting daily variation in staffing to improve understanding of the relationship between staffing and quality. They suggest that 2 facilities with the same average staffing achieve different quality of resident care and survey ratings in association with on the day-to-day variation in staffing. Measures of daily staffing may enhance the value of Nursing Home Care Compare for nursing homes and others engaged in quality improvement and consumers searching for high quality nursing homes.

## Key Points

**Question:**

Is daily variation in nursing home staffing associated with quality, and does it offer additional information to measures of average staffing levels?

**Findings:**

This quality improvement study of 13 339 nursing homes found that daily variation of staffing was significantly associated with the 5-Star Survey and Quality Measures rankings. There was little agreement between the variation and the average staffing measures on quality decile rankings of nursing homes, suggesting that staffing variation provides new quality improvement information.

**Meaning:**

These findings suggest that quality ranking of nursing homes and an understanding of the relationship between staffing and quality might be improved by adding daily variability measures to the Nursing Home Care Compare reports.

## Teaser

This quality improvement study examines daily variation in staffing, its association with quality, and whether daily variation provides information regarding quality ranking of nursing homes over and above the information provided by average staffing levels.

## Introduction

It is well established that direct-care staffing is fundamental to quality of nursing home care.^1^ A large evidence base suggests that the average level of staffing, in particular registered nurse (RN) and certified nurse aide (CNA) staffing, is associated with better performance on process quality measures, on-site survey scores, and resident outcome measures.^2,3,4,5,6,7^ Accordingly, established nursing home quality regulations, at both the federal level and in many states, include minimum nurse staffing standards.^8^ The COVID-19 pandemic underscored the role that staff played in mitigating outbreaks in nursing homes.^9^

Average staffing levels do not, however, fully capture the association of staffing to nursing home quality; ideally, one would know level of training, morale, and communication, among other things. For example, it has been recognized for decades that staff turnover—the frequency with which staff members quit or are terminated—is associated with quality.^10,11,12,13,14^ Due to prior data limitations, however, the Centers for Medicare & Medicaid Services (CMS) publishes only the case-mix adjusted average staffing levels^15^ in the Nursing Home Care Compare (NHCC) report card.^16^

One crucial phenomenon that has not been investigated to date is the day-to-day variation, or instability, of staffing levels. There have been recent calls to report weekend and weekday staffing separately in recognition that many nursing homes have significantly lower staffing on weekends.^17^ However, daily variation in staffing may extend beyond the difference between weekends and weekdays, as some nursing homes may have unstable staffing even during the week. Clearly, large fluctuations in numbers of staff may not be conducive to high-quality care, and measuring only average staffing levels may mask dangerously low levels on some days.

In this article, we examine daily variation in staffing using 3 possible measures. We take an initial look at these measures and explore the hypothesis that daily variation is negatively associated with quality for all Medicare and Medicaid–certified nursing homes nationally. To assess whether daily variation in staffing should be added to Care Compare, we also examine the hypothesis that daily variation provides information about the quality ranking of nursing homes over and above the information provided by average staffing levels.

## Methods

The study was approved by the University of California, Irvine, institutional review board. This report follows the Standards for Quality Improvement Reporting Excellence (SQUIRE) guidelines.

### Data Sources and Study Variables

The study included all 14 499 Medicare and Medicaid–certified nursing homes nationally with full year Payroll Based Journal (PBJ) data in 2017 and 2018. The PBJ data, collected by CMS, report the number of hours worked by each staff person each day, including contracted staff, for every facility. This data set also reports the resident census daily, calculated by CMS, based on the Minimum Data Set. We used these data to create variables measuring the daily number of RN and CNA hours per resident-day.

We used the Medicare provider number to merge these data at the facility level with the Medicare Cost Reports and NHCC, which provided information about nursing home characteristics. We excluded facilities with missing data. The final sample included 13 295 facilities (91.7%) for the RN analyses and 13 339 (92.0%) for the CNAs analyses.

### Variables

#### Variables Measuring Average Staffing and Daily Variation

**Figure 1.**
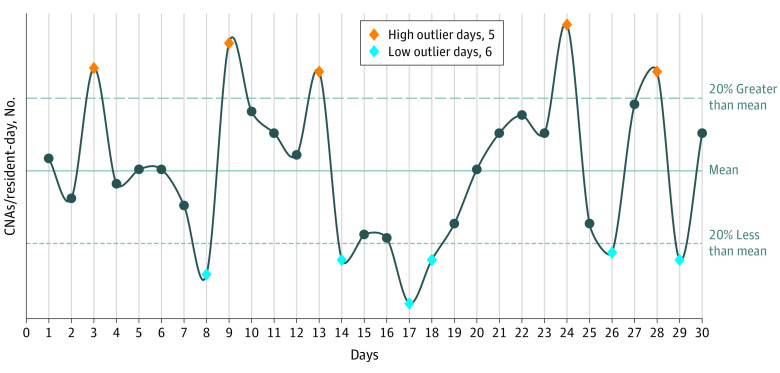
Example Calculation of Total Outlier Days and Low Outlier Days This hypothetical example presents daily fluctuations in certified nurse aide (CNA) staffing in a nursing home around its annual average CNA staffing level. The blue diamonds are the days that count toward the low outlier days variation measure, all less than 20% of the annual average line. In this example there are 6 low outlier days. The orange diamonds show the days exceeding the annual mean by more than 20%. In this example, there are 5 high outlier days. The total outlier days measure is the sum of the low and high outlier days; in this example, it is 11.

We defined 3 measures of daily variation for RN and CNA hours per resident-day. (We do not study licensed practical nurses [LPNs] because most prior studies^2,3,4,5^ failed to find an association between LPN average staffing levels and quality.) All 3 measures were measured at the facility level. The first, the coefficient of variation (COV), was defined as the ratio of the annual SD of the staffing hours per resident-day divided by its annual mean. The second, total outlier days (TOD), was defined as the number of days during the year in which staffing hours per resident-day deviated from the annual facility mean in either direction by at least 20%. The third, low outlier days (LOD), was defined as the number of days during the year in which staffing hours per resident-day were less than the annual facility mean by more than 20%. Figure 1 depicts measures 2 and 3. In sensitivity analyses, we tested measures defined on 30% thresholds. We also measured the average staffing hours per resident-day, the traditional staffing measure.

#### Variables Measuring Quality

We measured quality using 2 quality rating measures developed and published in NHCC by CMS and used since 2008 in the Quality Improvement Initiative and value-based programs.^15^ These are the 5-Star Survey and the 5-Star Quality Measures, which rate nursing homes on a scale from 1 (much below average) to 5 (much above average). These composite quality measures^18^ have been shown to inform behavior of patients^19,20^ and nursing homes.^21,22,23^

#### Other Nursing Home Characteristics

We controlled for other nursing home characteristics that were shown in previous studies to be associated with quality, including size (average annual resident census), case-mix-index (average resource utilization group–IV score),^24^ payer mix (percentage Medicaid, Medicare, and others),^25^ ownership,^24,26,27^ and chain affiliation.^25^ All variables were standardized (ie, expressed in terms of their Z statistic) to allow comparisons across variables.

### Statistical Analysis

We performed 2 types of analyses. The first was designed to assess the importance of daily variation in staffing by exploring its association with nursing home quality. We sought to understand characteristics of nursing homes associated with each of the daily variation measures and to examine associations with the 5-Star Quality Measures, controlling for facility characteristics. We estimated 6 regression models, 3 for RNs and 3 for CNAs. The dependent variables were the 6 combinations of RNs and CNAs with the 3 variation measures: COV, TOD, and LOD. All models had the same nursing home characteristics and quality measures as independent variables and included state fixed effects. We estimated generalized estimating equations with robust standard errors clustered at the facility level, using Stata version 16.1 (StataCorp). Statistical significance was set at *P* < .05, and all tests were 2-tailed.

The second set of analyses were designed to answer the following questions: does daily variation reveal new information not already captured by measures of average staffing? Would considering information about variation change nursing home rankings? To address these, we sorted nursing homes into deciles, one based on their average staffing and one based on their daily variation measures, from high to low. We created six 10 × 10 tables of matched nursing homes based on their average staffing and daily variation decile assignment (3 tables for RN and 3 for CNA variation measures). To assess levels of agreement, we report weighted κ for each of the 6 tables and present 1 table as an example. The degree of agreement between the average staffing measures and the variation measure in each table was measured by the weighted κ statistic,^28^ which considers the number of nursing homes agreement and disagreement. The weights reflect the distance of each nursing home from agreement, with nursing homes further away from agreement given decreasing weight compared with nursing homes closer to agreement. Visually, the increasing disagreement of the 2 measures regarding quality is represented by the distance from the diagonal. The κ ranges between 0 and 1, with 0 indicating no agreement and 1 indicating complete agreement. κ levels of 0.75-0.80 have been suggested as indicating agreement.^29,30^

## Results

Of the 13 339 nursing homes in the study, the mean (SD) number of residents per day for the year was 89.87 (51.43) (Table 1). A total of 2551 facilities (19%) were nonprofit, and 9476 (71%) were for profit, with 7727 (58%) chain affiliated. A mean (SD) 14% (13%) of residents were Medicare beneficiaries, and 55% (26%) were Medicaid beneficiaries.

**Table 1.** Descriptive Statistics

Characteristic	Sample		*P* values for difference
	Analysis	Excluded	
**Daily variation in staffing measures**			
Coefficient of variation			
RNs			
Nursing homes, No.	13 295	1157	.02
Mean (SD)	0.50 (0.58)	0.55 (0.75)	
CNAs			
Nursing homes, No.	13 339	1156	<.001
Mean (SD)	0.13 (0.11)	0.19 (0.17)	
**Total outlier days**			
RNs			
Nursing homes, No.	13 295	1157	.003
Mean (SD)	219.83 (68.58)	213.43 (81.62)	
CNAs			
Nursing homes, No.	13 339	1156	<.001
Mean (SD)	44.26 (45.20)	77.80 (69.03)	
**Low outlier days**			
RNs			
Nursing homes, No.	13 295	1157	.43
Mean (SD)	115.93 (45.39)	114.80 (54.90)	
CNAs			
Nursing homes, No.	13 339	1156	<.001
Mean (SD)	22.37 (23.71)	39.97 (39.40)	
**Average staffing measures**			
Hours per resident-day			
RN			
Nursing homes, No.	13 339	1160	<.001
Mean (SD)	0.41 (0.29)	0.93 (1.05)	
CNA			
Nursing homes, No.	13 339	1160	<.001
Mean (SD)	2.16 (0.49)	2.48 (0.73)	
**Quality measures from nursing home compare**			
Quality of Resident Care 5-Star Rating			
Nursing homes, No.	13 339	1053	<.001
Mean (SD)	3.87 (1.07)	3.73 (1.23)	
Survey 5-Star Rating			
Nursing homes, No.	13 339	1055	<.001
Mean (SD)	2.79 (1.19)	3.26 (1.24)	
**Other facility characteristics**			
Residents per day for the year			
Nursing homes, No.	13 339	1160	<.001
Mean (SD)	89.87 (51.43)	59.60 (58.84)	
Case-mix index			
Nursing homes, No.	13 339	94	.001
Mean (SD)	2.81 (0.30)	2.91 (0.35)	
Percentage of patients receiving Medicare			
Nursing homes, No.	13 339	123	<.001
Mean (SD)	13.94 (12.77)	30.98 (25.78)	
Percentage of patients receiving Medicaid			
Nursing homes, No.	13 339	123	<.001
Mean (SD)	54.63 (25.85)	33.73 (27.78)	
Percentage of patients with other payer			
Nursing homes, No.	13 339	123	.06
Mean (SD)	31.43 (22.65)	35.30 (21.48)	
Facility part of a chain			
Nursing homes, No.	13 339	123	.06
Yes, No. (%)	7727 (57.93)	61 (49.59)	
**Ownership**			
Nursing homes, No.	13 339	1160	<.001
For profit, No. (%)	9476 (71.04)	341 (29.40)	
Nonprofit, No. (%)	2551 (19.12)	507 (43.71)	
Government, No. (%)	520 (3.90)	234 (20.17)	
More than 1 type	792 (5.94)	78 (6.72)	
Hospital based			
Nursing homes, No.	13 339	1160	<.001
No. (%)	67 (0.50)	597 (51.47)	

Abbreviations: CNAs, certified nurse aide; RNs, registered nurses.

### Distribution of the Daily Variation Measures

These measures varied significantly across nursing homes. The mean (SD) COV was 0.50 (0.58) for RNs and 0.13 (0.11) for CNAs. The 10th to 90th percentile range for RNs (denoted henceforth as 10/90 range) was wide, at 0.22 to 0.82, and narrower for CNAs, at 0.07 to 0.20. The mean TOD measure for RNs was 220 (SD, 69; 10/90 range, 124-314) and, for CNAs, 44 (SD, 45; 10/90 range, 2-106). The mean LOD measure for RNs was 116 (SD, 45; 10/90 range, 62-168) and, for CNAs, 22 (SD, 24; 10/90, 0-54).

### Nursing Homes’ Characteristics Associated With the Daily Variation Measures

Table 2 presents regression models showing associations between the RN and CNA variability measures and facility characteristics. We present the results in terms of the average marginal effect (AME) of each characteristic. The AME is the average effect size of an increase of the characteristic by 1 SD. For example, an increase of 1 SD in the case-mix index was associated with a decrease of 0.023 in the COV, 3.67 in TOD, and 2.27 in LOD for RNs. This allows comparison of the associations (coefficients) across variables.

**Table 2.** Characteristics of Nursing Homes Associated With Daily Variation Measures^a^

Variable	COV				TOD				LOD			
	For RNs		For CNAs		For RNs		For CNAs		For RNs		For CNAs	
	Coefficient (95% CI)	*P* value	Coefficient (95% CI)	*P* value	Coefficient (95% CI)	*P* value	Coefficient (95% CI)	*P* value	Coefficient(95% CI)	*P* value	Coefficient (95% CI)	*P* value
CMS 5-Star ranking												
Quality Measures	−0.014 (−0.021 to −0.007)	<.001	−0.004 (−0.006 to −0.003)	<.001	−3.79 (−4.59 to −2.99)	<.001	−2.52 (−3.08 to −1.96)	<.001	−2.46 (−3.03 to −1.88)	<.001	−1.29 (−1.58 to −0.99)	<.001
Survey	−0.026 (−0.033 to −0.019)	<.001	−0.006 (−0.007 to −0.004)	<.001	−5.10 (−5.97 to −4.23)	<.001	−4.16 (−4.77 to −3.55)	<.001	−3.04 (−3.65 to −2.44)	<.001	−1.97 (−2.29 to −1.65)	<.001
Case mix	−0.023 (−0.034 to 0.012)	<.001	−0.002 (−0.005 to −0.002)	.01	−3.67 (−4.67 to −2.68)	<.001	−0.61 (−1.28 to 0.06)	.07	−2.27 (−2.97 to −1.57)	<.001	−0.37 (−0.74 to 0.00)	.05
Payers												
Medicaid	[Reference]	NA	[Reference]	NA	[Reference]	NA	[Reference]	NA	[Reference]	NA	[Reference]	NA
Medicare	−0.027 (−0.033 to −0.020)	<.001	0.004 (0.001 to 0.006)	.002	−4.90 (−5.95 − 3.84)	<.001	1.41 (0.84 to 1.97)	<.001	−2.91 (−3.64 to −2.18)	<.001	0.62 (0.31 to 0.94)	<.001
Other payers	−0.013 (−0.021 to −0.005)	.002	0.002 (−0.000 to 0.003)	.05	−2.77 (−3.72 to −1.82)	<.001	0.71 (0.08 to 1.35)	.03	−1.67 (−2.35 to −1.00)	<.001	0.22 (−0.13 to 0.57)	.22
Part of a chain	−0.016 (−0.033 to 0.001)	.06	−0.008 (−0.011 to −0.005)	<.001	−5.48 (−7.44 to −3.53)	<.001	−5.16 (−6.49 to −3.83)	<.001	−3.02 (−4.36 to −1.69)	<.001	−2.45 (−3.14 to −1.76)	<.001
Ownership												
For profit	[Reference]	NA	[Reference]	NA	[Reference]	NA	[Reference]	NA	[Reference]	NA	[Reference]	NA
Nonprofit	−0.015 (−0.042 to 0.012)	.28	−0.003 (−0.007 to 0.001)	.16	−10.35 (−12.93 to −7.77)	<.001	−5.08 (−6.72 to −3.44)	<.001	−5.80 (−7.54 to −4.06)	<.001	−2.62 (−3.50 to −1.74)	<.001
Government	−0.043 (−0.066 to −0.020)	<.001	−0.001 (−0.008 to 0.007)	.89	−9.57 (−13.98 to −5.15)	<.001	−4.51 (−7.09 to −1.93)	<.001	−7.17 (−10.01 to −4.32)	<.001	−2.36 (−3.72 to −1.00)	<.001
Other	−0.020 (−0.042 to 0.001)	.61	−0.003 (−0.007 to 0.001)	.17	−5.43 (−9.00 to −1.86)	.003	−1.89 (−4.21 to 0.43)	.11	−2.78 (−5.24 to −0.32)	.03	−1.02 (−2.21 to 0.17)	.09
Hospital based	−0.051 (−0.097 to −0.004)	.03	0.002 (−0.012 to 0.016)	.80	−2.11 (−13.34 to 9.11)	.71	0.980 ((−6.67 to 6.84))	.09	−2.78 (−9.68 to 4.12)	.43	1.26 (−2.86 to 5.39)	.55
Resident annual mean census	−0.127 (−0.140 to −0.114)	<.001	−0.025 (−0.027 to −0.023)	<.001	−27.28 (−28.94 to −25.61)	<.001	−21.73 (−23.01 to −20.45)	<.001	−16.92 (−18.01 to −15.82)	<.001	−10.15 (−10.82 to −9.47)	<.001

Abbreviations: CMS, Centers for Medicare & Medicaid Services; CNAs, certified nurse aide; COV, coefficient of variation; LOD, low outlier days; RNs, registered nurses; TOD, total outlier days.

^a^ Coefficients are reported as average marginal effects, and all continuous variables are standardized. All models include state fixed effects. The sample size for RNs was 23 398, with 13 295 facilities. The sample size for CNAs was 23 503, with 13 339 facilities.

#### Quality Measures

We measured the associations with the 5-Star Survey and the 5-Star Quality Measures rankings of the NHCC. Both were significantly and negatively associated with all the variation measures for both RNs and CNAs (Quality Measures: COV among RNs, −0.014 [95% CI, −0.021 to −0.007]; *P* < .001; COV among CNAs: −0.004 [95% CI, −0.006 to −0.003]; *P* < .001; TOD among RNs, −3.79 [95% CI, −4.59 to −2.99]; *P* < .001; TOD among CNAs, −2.52 [95% CI, −3.08 to −1.96]; *P* < .001; LOD among RNs, −2.46 [95% CI, −3.03 to −1.88]; *P* < .001; LOD among CNAs, −1.29 [95% CI, −1.58 to −0.99]; *P* < .001; Survey rankings: COV among RNs,−0.026 [95% CI, −0.033 to −0.019]; *P* < .001; COV among CNAs: −0.006 [95% CI, −0.007 to −0.004]; *P* < .001; TOD among RNs, −5.10 [95% CI, −5.97 to −4.23]; *P* < .001; TOD among CNAs, −4.16 [95% CI, −4.77 to −3.55]; *P* < .001; LOD among RNs, −3.04 [95% CI, −3.65 to −2.44]; *P* < .001; LOD among CNAs, −1.97 [95% CI, −2.29 to −1.65]; *P* < .001). The associations for RNs were higher than the those for CNAs, as were the associations for the Survey rankings compared with the Quality Measure rankings.

#### Ownership Type

We measured the association of the daily variation measures with nonprofit, government, and other ownership, compared with for-profit nursing homes. These results were mixed. They were significant for the outlier days measures with an effect size that was twice as large for RNs than CNAs for both nonprofit and government-owned facilities vs for-profit facilities (Table 2). They were not generally significant for the COV measure and for other ownership. This suggests that for-profit facilities tend to have less stable staffing. We note that the low variability of nonprofit nursing homes’ staffing, for both RNs and CNAs, relative to for-profit nursing homes, exhibited the second largest effect size of all the associations in the models.

#### Chain Ownership

Being part of a chain was significantly and negatively associated with all measures of variation for both RNs and CNAs (Table 2). However, it was not associated with the COV for RNs.

#### Payer Mix

We measured the association with respect to Medicare and other payers relative to Medicaid. A higher percentage of Medicare beneficiaries was significantly associated with lower variation of RNs but higher variation of CNAs compared with Medicaid (Table 2). A similar pattern existed for other payers, although it was not as significant for the CNAs. The associations were larger by approximately 50% for Medicare for variation among RNs variation and for the other payer among CNAs.

#### Case-Mix

Higher case-mix was negatively and significantly associated with all 3 measures (Table 2). This suggests that nursing homes with a higher case-mix index tend to have more stable staffing mix.

#### Hospital Based Nursing Home

This variable was not significantly associated with any of the variation measures.

#### Size

Size was measured as the annual average resident census and included in the model as a linear and square term to allow for nonlinearity in scale. It was included not just as a facility characteristic of interest but also because it can affect the variation measures statistically. For example, if 1 person is absent in a nursing home employing 100 people, this facility would miss only 1% of its employees; another facility employing only 10 people would miss 10% of its employees, making smaller facilities naturally less stable. This variable, therefore, plays multiple roles and should be interpreted carefully. As expected, size was significantly and negatively associated with staffing variation (Table 2); larger facilities scored lower on all of the variability measures, and it had the largest coefficients compared to all other characteristics.

eTable 1 in the Supplement presents results for the daily variability measures defined with the threshold set at 30%. These results were similar to those with the 20% threshold.

### Quality Ranking by Daily Staffing Variation Compared With Ranking by Average Staffing Levels

**Figure 2.**
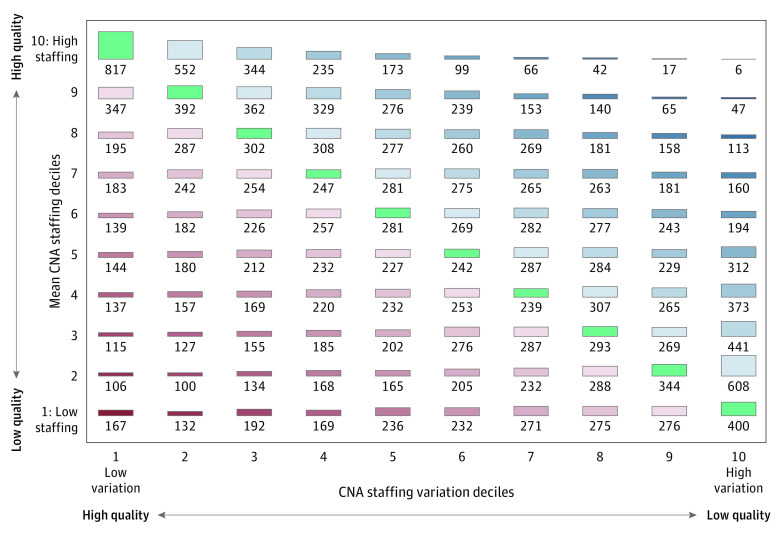
Mean Certified Nurse Aide (CNA) Staffing vs CNA Staffing Daily Variation This table presents the number of nursing homes nationally ranked into quality deciles based on (1) mean CNA staffing (y-axis) and (2) daily variation in CNA staffing (x-axis). The numbers on the diagonal are the numbers of nursing homes for which the 2 measures agree on the decile ranking. The cells in blue are those for which mean quality rankings are greater than variability ranking. The cells in red are those for which the variability quality rankings are greater than the mean quality ranking.

Figure 2 presents an example of a 10 × 10 table, 1 of 6 we created, that classified each nursing home based on the average staffing measure and 1 of the variation measures. It shows the classification of nursing homes based on LOD among CNAs. Five additional tables (not presented) were created for CNAs and RNs with all other variation measures. All nursing homes in green on the diagonal are those in which the average staffing measure and the variation measure classify the nursing home to the same quality decile. Nursing homes falling into off-diagonal cells are those for which the 2 measures disagree; the further from the diagonal, the greater the disagreement.

 The values of the weighted κ we measured were all less than 0.6, except for the COV for RNs, which was 0.626 (95% CI, 0.617-0.634). The other values were as follows: TOD for RNs, 0.537 (95% CI, 0.529-0.546); LOD for RNs, 0.513 (95% CI, 0.505-0.521); COV for CNAs, 0.234 (95% CI, 0.226-0.242); TOD for CNAs, 0.250 (95% CI, 0.242-0.258); LOD for CNAs, 0.252 (95% CI, 0.243-0.260). These values indicate 2 things: first, there is little agreement between the classification of average staffing measures and the 3 variation measures into high and low quality, and second, the disagreement is much larger for the CNA measures than for the RN measures. The minimal disagreement can be observed visually in Figure 2. The diagonal cells include only about one-third of the nursing homes. The other two-thirds populate all off-diagonal cells, including cells far from the diagonal, indicating substantial disagreement between the two measures. eTable 2 in the Supplement reports weighted κ values for the variation measures defined with thresholds at 30%, which are similar.

## Discussion

This article examined the possibility that information about daily variation in nursing home staffing might add to our understanding of quality above and beyond average levels of staffing. It studied this question in 3 ways and found that daily variation seems to matter to quality. It found that (1) there is a significant association between 3 measures of daily variation and 2 widely accepted measures of quality, the 5-Star Survey and the 5-Star Quality Measures ranking; (2) there is a significant association between staffing variability and nursing home ownership, which has been shown to be associated with higher quality (eg, nonprofit nursing homes that have been shown to have higher levels of quality^27^ have lower levels of staffing daily variation compared with for-profit nursing homes); and (3) there is little agreement between nursing home quality decile ranking based on average staffing and variation in daily staffing levels, suggesting that the variation measures offer information above and beyond that provided by the traditional average staffing measure.

The disagreement about ranking of nursing homes between the measure based on average staff levels and the variation measures has implications for public report cards, consumer choice of nursing homes, contracting with payers, and pay-for-performance systems. Consider a consumer choosing a nursing home in his or her town and having the following 2 nursing homes to choose from in Figure 2: a facility ranked 9 for staffing and 10 for variability, indicating a very large staff with high variability, and another facility ranked 5 for staffing and 1 for variability, indicating a much smaller staff but low variability. A choice based on staffing level alone will obviously be to select the first facility. But is this the best choice? We do not have the answer at this time. More research is required to answer this question because it is a tradeoff, like other tradeoffs between quality measures, such as those between staffing levels and other 5-Star Quality Measures, 5-Star Survey ranking, and other reported quality measures.^31^ However, currently, consumers may not be aware that nursing homes differ substantially along the dimension of staffing variability and that daily variability is associated with the quality of care they might receive.

What might be the underlying processes leading to an association between variation in staffing and patient health outcomes? The answer to this question is both clinical and organizational. From a clinical care perspective, residents benefit from access to regular, as opposed to inconsistent, nursing assessment and care. For example, wide variation in staffing levels might lead to low reliability in the early detection of changes in symptoms that is important for triggering evaluations and interventions critical to preventing worsening health status. From an organizational perspective, the absence of stable staffing inputs impedes management’s ability to create a high-reliability organization with consistent, safe workflows. Everyday tasks, such as medication administration and monitoring, can be adversely affected by both inadequate staffing and a lack of stability in staffing availability. During low staffing days, when residents do not receive needed care, they are more likely to develop various conditions, such as pressure injuries, because staffing was not sufficient to rotate them in bed; exacerbation of wounds when staff is not available to change dressings in a timely fashion; falls with injuries without consistent daily attention to anticipating needs, such as requiring assistance in getting to the bathroom. Most of these consequences of short staffing cannot be fixed by additional staff on other days: more turning or toileting on extra-staffing days cannot eliminate the fall or the wound development that occurred when understaffed.

### Limitations

This study has limitations. We note our inability to adjust the variability measures for daily fluctuations in case-mix. If these are frequent and substantial enough to lead nursing homes to adjust staffing, then the variability measures should ideally account for them. We also note that our sample excluded 8% of nursing homes. The excluded facilities tended to be hospital based, smaller, and caring mostly for Medicare beneficiaries. Our findings may not generalize to them.

There is also a possibility that daily staffing variability and turnover, which has also been recognized as associated with patient outcomes,^11,13^ are themselves related, at least to some degree. While daily staffing variation is likely due to absenteeism, poor scheduling, and labor shortages, it may also be driven by some of the same mechanism that have been identified for turnover, including work conditions,^32^ low wages,^33^ and others.^34,35^ This study has not addressed the unique contribution of each phenomenon to quality and outcomes that should be addressed in future work.

## Conclusions

The availability of the PBJ data as of 2017 opens new opportunities to study the associations between staffing and quality from perspectives that were not possible before and to apply new knowledge to policy and quality standards. In this study, we took a first step in this direction, finding that daily variation in staffing levels was associated with nursing home quality. Considering variation and not just levels of staffing can offer new perspectives on staffing management in nursing homes and potentially improve quality. More work is required to understand daily variation in the broader context of goals for staffing and to determine the best, most valid, and most informative measures.

## Notes

Supplement. **eTable 1.** Characteristics of Nursing Homes Associated With Daily Variation Measures Sensitivity Analysis: Total Outlier Days/Low Outliers Days at Threshold of 30% **eTable 2.** Weighted κ Statistic for Agreement Between Mean Staffing and Daily Staffing Variation: Sensitivity Analysis With Measures Based on 30% Threshold **eTable 3.** Characteristics of Nursing Homes Associated with CMS 5-Star Ranking: Staffing Sensitivity Analysis
