# Supplementary material for: Daily Variation in Nursing Home Staffing and Its Association With Quality Measures
Source: JAMA Netw Open. 2022 Mar 14;5(3):e222051. doi: 10.1001/jamanetworkopen.2022.2051 (PMC9907340; doi:10.1001/jamanetworkopen.2022.2051)
Supplement: Supplement. — eTable 1. Characteristics of Nursing Homes Associated With Daily Variation Measures Sensitivity Analysis: Total Outlier Days/Low Outliers Days at Threshold of 30% eTable 2. Weighted κ Statistic for Agreement Between Mean Staffing and Daily Staffing Variation: Sensitivity Analysis With Measures Based on 30% Threshold eTable 3. Characteristics of Nursing Homes Associated with CMS 5-Star Ranking: Staffing Sensitivity Analysis [file jamanetwopen-e222051-s001.pdf]

Supplemental Online Content

Mukamel DB, Saliba D, Ladd H, Konetzka RT. Daily variation in nursing home staffing and its association with quality measures. *JAMA Netw Open.* 2022;5(3):e222051. doi:10.1001/jamanetworkopen.2022.2051

eTable 1. Characteristics of Nursing Homes Associated With Daily Variation Measures Sensitivity Analysis: Total Outlier Days/Low Outliers Days at Threshold of 30%

eTable 2. Weighted κ Statistic for Agreement Between Mean Staffing and Daily Staffing Variation: Sensitivity Analysis With Measures Based on 30% Threshold

eTable 3. Characteristics of Nursing Homes Associated with CMS 5-Star Ranking: Staffing Sensitivity Analysis

This supplemental material has been provided by the authors to give readers additional information about their work.

© 2022 Mukamel DB et al. *JAMA Network Open.*

**eTable 1.** Characteristics of Nursing Homes Associated With Daily Variation Measures Sensitivity Analysis: Total Outlier Days/Low Outliers Days at Threshold of 30%				
**Dependent Variables** / **Independent Variables**	**Total Outlier Days**		**Low Outlier Days**	
	**RNs Daily Staffing Variation Measures**	**CNAs Daily Staffing Variation Measures**	**RNs Daily Staffing Variation Measures**	**CNAs Daily Staffing Variation Measures**
CMS 5-Star, Quality	-4.37<br><0.001	-1.25<br><0.001	-2.70<br><0.001	-0.66<br><0.001
CMS 5-Star, Survey	-5.77<br><0.001	-1.91<br><0.001	-3.23<br><0.001	-0.89<br><0.001
Average case mix (RUGS IV)	-4.32<br><0.001	-0.21<br>0.344	-2.64<br><0.001	-0.16<br>0.232
Payers:				
Medicaid (Reference)				
Percent Medicare patients	-6.29<br><0.001	0.81<br><0.001	-3.73<br><0.001	0.31<br>0.004
Percent other payers	-3.33<br><0.001	0.48<br>0.021	-1.96<br><0.001	0.13<br>0.279
Nursing Home is part of a chain	-6.38<br><0.001	-2.41<br><0.001	-3.72<br><0.001	-1.16<br><0.001
Ownership:				
For profit (Reference)				
Nonprofit ownership	-11.95<br><0.001	-1.40<br>0.009	-6.61<br><0.001	-0.49<br>0.116
Government ownership	-10.38<br><0.001	-2.07<br>0.006	-7.36<br><0.001	-0.81<br>0.062
Other ownership	-6.55<br>0.001	-0.92<br>0.181	-3.58<br>0.008	-0.24<br>0.547
Nursing Home is hospital based	-4.62<br>0.435	0.70<br>0.741	-4.34<br>0.215	0.81<br>0.513
Size: Resident annual average census	-34.20<br><0.001	-10.75<br><0.001	-19.57<br><0.001	-4.72<br><0.001
Sample size	23,398	23,503	23,398	23,503
Number of facilities	13,295	13.339	13.295	13.339

Note: Coefficients are reported as average marginal effects and all continuous variables are standardized. P values in parentheses.

© 2022 Mukamel DB et al. *JAMA Network Open.*

**eTable 2**. Weighted κ Statistic for Agreement Between Mean Staffing and Daily Staffing Variation: Sensitivity Analysis With Measures Based on 30% Threshold		
	RNs	CNAs
	Weighted Kappa	Weighted Kappa
Total Outlier Days (TOD)	0.565	0.217
Low Outlier Days (LOD)	0.530	0.226

© 2022 Mukamel DB et al. *JAMA Network Open.*

eTable 3. Characteristics of Nursing Homes Associated with CMS 5-Star Ranking: Staffing Sensitivity Analysis						
**Dependent Variables** / **Independent Variables**	**Coefficient of Variation (COV)**		**Total Outlier Days (TOD)**		**Low Outlier Days (LOD)**	
	**RNs Daily Variation Measure**	**CNAs Daily Variation Measure**	**RNs Daily Variation Measure**	**CNAs Daily Variation Measure**	**RNs Daily Variation Measure**	**CNAs Daily Variation Measure**
CMS 5-Star ranking, QMs	-0.012<br><0.001	-0.004<br><0.001	-3.43<br><0.001	-2.46<br><0.001	-2.19<br><0.001	-1.26<br><0.001
CMS 5-Star ranking, Survey	-0.019<br><0.001	-0.006<br><0.001	-4.22<br><0.001	-3.98<br><0.001	-2.40<br><0.001	-1.89<br><0.001
CMS 5-Star ranking, Staffing	-0.090<br><0.001	-0.006<br>0.219	-14.44<br><0.001	-2.06<br><0.001	-9.67<br><0.000	-1.07<br><0.001
Average case mix (RUGS IV)	-0.027<br><0.001	-0.002<br>0.014	-4.44<br><0.001	-0.70<br>0.040	-2.78<br><0.001	-0.41<br>0.027
Payers:						
Medicaid (Reference)						
Percent Medicare patients	-0.012<br><0.001	0.004<br>0.001	-2.54<br><0.001	1.74<br><0.001	-1.32<br><0.001	0.80<br><0.001
Percent other payers	-0.002<br>0.514	0.002<br>0.044	-1.07<br>0.023	1.00<br>0.002	-0.49<br><0.001	0.37<br>0.042
Nursing Home is part of a chain	-0.020<br>0.010	-0.008<br><0.001	-6.08<br><0.001	-5.28<br><0.001	-3.43<br><0.001	-2.51<br><0.001
Ownership:						
For-Profit (Reference)						
Nonprofit ownership	0.032<br>0.026	-0.002<br>0.311	-2.88<br>0.029	-4.07<br><0.001	-0.77<br>0.392	-2.09<br><0.001
Government ownership	0.003<br>0.794	+0.000<br>0.978	-3.50<br>0.124	-3.76<br>0.005	-3.15<br>0.032	-1.97<br>0.006
Other ownership	0.004<br>0.741	-0.003<br>0.226	-1.62<br>0.377	-1.42<br>0.237	-0.31<br>0.809	-0.77<br>0.209
Nursing Home is hospital based	-0.035<br>0.158	0.002<br>0.771	-1.52<br>0.785	0.32<br>0.926	-1.97<br>0.578	1.40<br>0.511
Size: Resident annual average census	-0.137<br><0.001	-0.025<br><0.001	-29.28<br><0.001	-22.12<br><0.001	-18.23<br><0.001	-10.34<br><0.001
Sample size	23,398	23,503	23,398	23,503	23,398	23,503
Number of facilities	13,295	13,339	13,295	13,339	13,295	13,339

© 2022 Mukamel DB et al. *JAMA Network Open.*
